# Understanding Market Size and Reporting Gaps for Paediatric TB in Indonesia, Nigeria and Pakistan: Supporting Improved Treatment of Childhood TB in the Advent of New Medicines

**DOI:** 10.1371/journal.pone.0138323

**Published:** 2015-10-13

**Authors:** Renia Coghlan, Elizabeth Gardiner, Farhana Amanullah, Chikwe Ihekweazu, Rina Triasih, Malgorzata Grzemska, Charalambos Sismanidis

**Affiliations:** 1 TESS Development Advisors, Geneva, Switzerland; 2 Consultant, New York, New York, United States of America; 3 Paediatric TB Programme, The Indus Hospital, Karachi, Pakistan; 4 EpiAfric, Abuja, Nigeria; 5 Department of Paediatrics, Dr. Sardjito Hospital/Faculty of Medicine, Universitas Gadjah Mada, Yogyakarta, Indonesia; 6 Global TB Programme, World Health Organization, Geneva, Switzerland; Fondazione IRCCS Ca' Granda Ospedale Maggiore Policlinico, Università degli Studi di Milano, ITALY

## Abstract

**Objective of the Study:**

We sought to understand gaps in reporting childhood TB cases among public and private sector health facilities (dubbed “non-NTP” facilities) outside the network of national TB control programmes, and the resulting impact of under-reporting on estimates of paediatric disease burden and market demand for new medicines.

**Methodology:**

Exploratory assessments were carried out in Indonesia, Nigeria and Pakistan, reaching a range of facility types in two selected areas of each country. Record reviews and interviews of healthcare providers were carried out to assess numbers of unreported paediatric TB cases, diagnostic pathways followed and treatment regimens prescribed.

**Main Findings:**

A total of 985 unreported diagnosed paediatric TB cases were identified over a three month period in 2013 in Indonesia from 64 facilities, 463 in Pakistan from 35 facilities and 24 in Nigeria from 20 facilities. These represent an absolute additional annualised yield to 2013 notifications reported to WHO of 15% for Indonesia, 2% for Nigeria and 7% for Pakistan. Only 12% of all facilities provided age and sex-disaggregated data. Findings highlight the challenges of confirming childhood TB. Diagnosis patterns in Nigeria highlight a very low suspicion for childhood TB. Providers note the need for paediatric medicines aligned to WHO recommendations.

**Conclusion: How Market Data Can Support Better Public Health Interventions:**

This study emphasises the impact of incomplete reporting on the estimation of disease burden and potential market size of paediatric TB medicines. Further studies on “hubs” (facilities treating large numbers of childhood TB cases) will improve our understanding of the epidemic, support introduction efforts for new treatments and better measure markets for new paediatric medicines.

## Introduction

Childhood TB has long been a neglected health problem, with little national or international attention, especially for those under five years [1–6]. Global interest has been re-ignited [7–8] with developments such as the World Health Organization’s (WHO) first estimates in 2012 of global TB disease burden among children under 15 years [9], the updated estimates in 2013 [10], the launch of the Roadmap for Childhood TB in 2013 [11], and WHO’s 2^nd^ edition of the Childhood TB Guidelines [12].

Despite increased awareness and interest in paediatric TB data, well-described difficulties remain in the estimation of burden of childhood TB [9–10, 13–14]. This is mainly due to the lack of age-specific national TB surveillance data [9–11], difficulties with diagnosis [15–18] and gaps in reporting of childhood TB cases from both public and private sector facilities outside the network of the National TB Programmes (henceforth referred to as non-NTP facilities) [10, 19–22]. The non-NTP sector here includes both not-for-profit and for-profit healthcare facilities and medicine-dispensing outlets (e.g. pharmacies and drug shops) involved in the diagnosis or treatment of paediatric TB cases. This includes public sector facilities not under the management of the National TB Programmes.

Due to the lack of a simple, precise, point-of-care tool, the diagnosis of TB in children is often based on a combination of clinical symptoms and chest X-ray, especially in high burden countries [15–18]. Treatment is complicated by the absence of appropriately formulated, paediatric-specific medicines that comply with current WHO treatment guidelines [23–25]. In addition, symptoms of childhood TB may be masked by other diseases such as pneumonia, a relatively common childhood ailment [16–17]. Suspicion for childhood TB may be lower than for adults, increasing the potential for inappropriate or late treatment-seeking, under- or mis-diagnosis [21].

Gaps in reporting, particularly from non-NTP facilities, limit our understanding of the overall numbers of children diagnosed and treated in a country particularly when the private sector is a major provider of healthcare generally. In addition, such gaps make it unclear whether this sector plays an equally important role in the diagnosis and treatment of paediatric TB specifically [26–29]. Very few studies have been found focusing on the role of the non-NTP sector on pathways to treatment for paediatric TB [30].

Furthermore, no studies were found to have focused directly on the links between under-reporting from these sectors on disease burden estimates and market size for paediatric medicines. Thus, the link between disease burden, reporting rates and market size estimates for paediatric TB products remains unchartered territory.

As long as there is major uncertainty around paediatric TB burden numbers, market size and dynamics for paediatric tuberculosis medicines [30], new manufacturers will have little incentive to develop or market new appropriately formulated paediatric TB medicines, leading to a status quo of treatment options for children. This study, as part of a larger portfolio of studies by the TB Alliance and WHO, is part of a joint effort to address this status quo, and clearly indicates that the market potential for childhood treatment is larger than current public sector estimates.

## Methods

The study combined a review of patient records and interviews of non-NTP sector healthcare providers in Indonesia, Nigeria and Pakistan to understand gaps in the reporting of children seen in non-NTP facilities, to assess diagnostic and treatment pathways for paediatric TB cases, and to generate further information around the paediatric TB medicines market. Our study was designed to be exploratory, and aimed to identify areas of focus for further research.

Three parallel reviews were carried out across a two-month period (August and September 2013) using a common study protocol in the three countries.

The countries in which the study was implemented—Indonesia, Nigeria and Pakistan—were selected from those with the highest estimated disease burden due to TB, high private sector involvement in healthcare provision, a mix of high and low HIV prevalence, and suspicions of under-reporting from the private sector of paediatric TB [14–15, 27].

In the absence of official registers of facilities diagnosing and treating paediatric TB patients, the research teams first used a snowball sampling approach: a technique which uses non-probability sampling of experts or others to identify subjects (in this case facilities) which are hard to identify [31]. Using this method, the researchers identified facilities providing these services in two geographically defined zones in each country covering both urban and rural areas. The range of facilities visited, outlined in Table 1, included both traditional treatment centers as well as those that are not normally identified as places where paediatric TB cases are found, in order to understand the importance of different facility types as paediatric TB treatment hubs.

**Table 1:** Summary of Sites Visited, by Country and Facility Type.

	Indonesia	Nigeria	Pakistan
Army base hospital	2	0	1
Mother and child clinic	7	0	0
District hospital	5	0	0
Private clinic (outpatient)	6	0	0
Private hospital	15	8	7
Private children’s hospital	0	4	0
Private practice (paediatrician)	11	2	1
Private practice (general practitioner)	7	0	14
Private practice (midwife)	1	0	0
Faith-based hospital	9	3	0
Public hospital (inpatient)	1	3	3
Public hospital (outpatient)	0	5	0
Rural health center (NGO)	0	2	0
HIV treatment center	0	1	0
Pharmacy	0	8	2
Patent Medicine Vendor	0	4	0
Lab / diagnostic center	0	5	7
**Total**	**64**	**45**	**35**

Facilities were asked to provide records of paediatric patients with diagnosed TB covering the three months prior to the start of the study. Where records were available, they were reviewed to assess the numbers of paediatric TB cases identified, the tests ordered for diagnosis and the treatment regimen prescribed. As patient records were unavailable (did not exist or were not shared) in 88% of the facilities visited, semi-structured interviews of healthcare providers were carried out to complement the information extracted from the records. In addition to information on the numbers of children seen in each facility, diagnostic pathways and treatment preference, the interviews were also used to elicit information on provision of contact tracing and education on TB prevention. Results were compared across the three countries to identify common practices.

The assessment in Indonesia took place in two provinces, West Sumatra and Yogyakarta. A total of 64 facilities were visited, distributed over three rural and one urban districts in West Sumatra and four rural and one urban districts in Yogyakarta. Fifty-three of these facilities saw paediatric TB cases.

The Pakistan assessment included 35 facilities, nearly half of which were General Practitioners (family medicine doctors). The facilities were spread over three areas: Karachi, Islamabad and Rawalpindi, all representing urban areas. All the sites reported seeing paediatric TB patients.

The Nigerian study was carried out in one urban and one peri-urban location in Enugu and Lagos. A total of 45 facilities were visited, 20 of which saw paediatric TB patients. Unlike the studies in Asia, the Nigerian survey included a higher proportion of pharmacies and patent medicines vendors (drug shops), in line with common healthcare practice in that country [22]. Interviews in all three countries were carried out only in sites that confirmed that they saw paediatric TB patients.

### Ethical Approval

In discussions with the National TB Programmes (NTP) of each country, the studies were considered as part of the NTPs’ surveillance activities, supporting Public Private Mix (PPM) outreach activities (as described in the Global TB Report 2013, page 31) [10]. As such, ethical review was not required or sought. Permission for the study was granted by the NTP Manager in each country.

#### Indonesia

The survey team asked the facilities for information on the number of children treated as TB and other questions as per the survey questionnaire. In some of the facilities, the doctors showed 2–3 medical records to confirm that they use the Indonesian TB scoring system and to show how this was documented in the medical records. The team verified these on site during the interview and did not collect or record any individual patient data. A letter from the Indonesian TB Programme Manager at the national Ministry of Health confirmed that the survey was considered part of routine surveillance and exempting it from ethics clearance for that reason.

#### Pakistan

Individual patient records were not made available by the facilities and were not reviewed by the survey team. The team received verbal reports of numbers and answers to the interview questions during the interviews with the doctors. There were only 2 instances when the survey team looked at registers which carried de-identified data (not patient medical records): one register had patient ages and diagnoses, while the other held information on number of chest x-rays and TSTs done (among other tests) in children. Neither register had personally identifiable patient information.

#### Nigeria

The team did not have direct access to patient records. The teams provided the administrator / nurse / pharmacist or doctor with the questions. The facility contact point counted the information from the patient registers and provided the information to the survey team orally during the interview. The survey team then recorded the information provided on aggregated questionnaires (one per facility).

Information was mainly provided by the facility interviewee, except in 2 to 3 facilities in Indonesia as described above, and access to a small number of de-identified registers in Pakistan. This further highlights the challenge of confirming recording of data by the healthcare providers in relation to childhood TB. Data were not copied or removed from the facility: relevant data on the numbers of children treated and treatment pathways were collected through the interviews, aggregated and then recorded in Word document interview guides. Information on the demographics of the healthcare providers was limited to their specialty in order to identify the key types of provider seeing paediatric TB patients (e.g. paediatricians vs generalists). The healthcare providers were notified of the study, and shown an authorization letter provided by the Ministry of Health national TB control programme, in view of the study’s contribution to national TB surveillance activities.

The national lead investigators were: Dr Rina Triasih in Indonesia, Dr Chikwe Ihekweazu in Nigeria and Dr Farhana Amanullah in Pakisan. Renia Coghlan served as overall study principal investigator, was responsible for drafting the protocol, coordinating the study, multi-country analysis and reporting the findings.

## Results

### Case Identification and Reporting Gaps

Details on the types of facilities visited, total numbers of children seen by these facilities and numbers of paediatric TB cases identified are shown in Table 2. Based on the records available and provider interviews, a total of 985 diagnosed but unreported paediatric TB cases were identified for a three-month period in Indonesia in 53 facilities, 463 cases in Pakistan in 35 facilities and 24 cases in Nigeria in 20 facilities. Assuming similar treatment rates throughout the year and extrapolating these numbers to an annual basis, this limited number of facilities represents the equivalent of an annualised additional yield of new paediatric cases reported to WHO of 15% for Indonesia, 2% for Nigeria and 7% for Pakistan.

**Table 2:** Overview of findings: number of children seen in non-NTP facilities, diagnosed with suspect or confirmed tuberculosis, but not reported to NTP (by country, (May–July 2013).

	Indonesia	Nigeria	Pakistan
Nr Facilities identified and screened	64	45	35
Nr Facilities seeing paediatric TB patients	53	20	35
Total Nr Paediatric patients (all cause)	108,000	16,000	115,000
Nr additional paediatric TB patients unreported (limited nr sites, 3 month period)	985	24	463
Total nr new paediatric cases TB reported to WHO 2013	26,054	5,776	28,113
**Estimated** equivalent nr unreported / year (in sites surveyed: **NOT** equivalent to national)	3,940	96	1,852
Total nr of cases found compared in absolute nrs to annual reports to WHO (limited sites, 3 months)	3.5%	2%	4%
Annual additional cases found, as % relative to annual reports to WHO (estimates from the limited sites)	15%	2%	7%

Availability of high quality patient records was limited. Although all facilities in Indonesia were able to show the existence of patient records, patient records were available in only 50% of the 20 facilities in Nigeria, while no case records were made directly available in Pakistan. However, the records available were generally insufficiently detailed to verify the diagnosis and case type for paediatric TB cases. Age and sex data were available in only 12% of all facilities visited: eight of the 53 facilities in Indonesia, five of the 20 facilities in Nigeria and none of the facilities in Pakistan. Poor record keeping and reporting linkages were identified as key obstacles in understanding the scale of childhood TB in these high burden settings. As a result, analysis focused on a combination of patient record data and the findings from the healthcare provider interviews.

### Importance of Facility Type as Locus of Treatment

The research team wished to identify the relative importance of different facilities types for the diagnosis of childhood TB: i.e. did facilities which saw the most children also diagnose and treat the highest number of paediatric TB cases? A review of the total number of paediatric patients seen (all causes), indicating overall patient volume per facility, versus the number of paediatric TB cases seen show large numbers of children diagnosed with TB seen in non-NTP facilities in Indonesia (0.9% of all paediatric cases seen at these facilities) and Pakistan (0.4% of all paediatric cases seen), while not as many were found in Nigeria (0.2% of all paediatric cases seen). However, there was no relationship between overall patient volume and the number of TB patients identified at a facility level (see Table 3). Ten of the 53 facilities in Indonesia saw a total of 66% of the cases found, while just six of the 35 facilities interviewed in Pakistan treated 70% of all unreported cases identified in that country, indicating the existence of treatment hubs.

**Table 3:** Numbers of Paediatric Patients Estimated Per Facility Type and Numbers of Estimated New Paediatric TB Cases.

Facility types	Number of facilities	Total Number of paediatric patients seen (all causes)	Nr unreported diagnosed paediatric TB cases
**INDONESIA**			
Military hospital	2	800	20 to 40
Mother and child clinic	3	900 to 5,600	0 to 6
District hospital	5	1,100 to 2,800	16 to 133
Private clinic (outpatient only)	5	200 to 1,000	1 to 12
Private hospital (with inpatient)	15	240 to 3,120	3 to 50
Private practice (paediatrician)	10	230 to 2,280	1 to 35
Private practice (GP)	3	320 to 2,500	1 to 4
Private practice (midwife)	1	480	4–8
Faith-based hospital	8	400 to 4400	1 to 120
Public hospital	1	59,000	2
Pharmacy	0	N/A	N/A
Diagnostic center / Lab	0	N/A	N/A
**NIGERIA**			
Military hospital	0	N/A	N/A
Mother and child clinic	0	N/A	N/A
District hospital	0	N/A	N/A
Private clinic (outpatient only)	0	N/A	N/A
Private hospital (with inpatient)	8	100 to 300	6
Private children’s hospital	4	360 to 840	4
Private practice (paediatrician)	2	120 to 2250	2
Private practice (GP)	0	N/A	N/A
Private practice (midwife)	0	N/A	N/A
Faith-based or NGO hospital	4	360 to 3,870	1
Public hospital (inpatient)	4	150 to 4,000	0
Public hospital (outpatient)	5	300 to 1,350	0
Pharmacy	8	108 to 1,000	1
Patent Medicine Vendor	4	810	0
Diagnostic center / Lab	5	96 to 168	2
HIV treatment center	1	20,000	0
PAKISTAN			
Military hospital	1	7560	60
Mother and child clinic	0	N/A	N/A
District hospital	0	N/A	N/A
Private clinic (outpatient only)	0	N/A	N/A
Private hospital (with inpatient)	7	1,200 to 15,552	3 to 100
Private practice (paediatrician)	1	6,000	1
Private practice (GP)	14	575 to 7000	2 to 15
Private practice (midwife)	0	N/A	N/A
Faith-based hospital	0	N/A	N/A
Public hospital	3	360 to 36,000	12 to 30
Pharmacy	2	N/A	3 to 36
Diagnostic center / Lab	6	N/A	3 to 408

In Indonesia and Pakistan, paediatricians—both in hospitals and in private practice—were confirmed to be central to the diagnosis and treatment pathway. Most children were brought to a paediatrician as a first point of investigation. Many of the others were either referred to a paediatrician or returned to one for treatment. In addition, laboratories were a key center for diagnosis in Pakistan with 75% of all suspected cases sent to labs for testing (see Table 2). There was no dominant facility type identified in Nigeria.

### Diagnostic Pathway and Treatment Preferences

Understanding of the diagnostic pathway, through healthcare provider interviews, indicated that a wide variety of approaches were used in all three countries in addition to clinical symptoms (see Table 4). The combination of tests also varied significantly. On average, practitioners requested between 1–2 tests per child in Nigeria, 2–3 in Indonesia and 3–4 tests in Pakistan. Results of the interviews from Nigeria suggest limited suspicion of TB in children when they present to healthcare facilities: children are therefore rarely considered for testing and the correct diagnosis is often missed. National TB guidelines and scorecards exist in all three countries to help guide clinicians in diagnosis, but were not widely used by most of the practitioners interviewed. In many cases, healthcare providers preferred to refer patients on to a paediatrician at a hospital or in private practice for further diagnosis.

**Table 4:** Diagnostic Tests requested by healthcare practitioners when faced with a suspect case of paediatric TB (several tests may be requested for one child).

Diagnostic Approach: Frequency Mentioned (total nr of facilities seeing paeds TB pts)	Clinical Symptoms (Clinical Symptoms; Cough +2 weeks)	Radiological Tests (Chest X-Ray)	Supportive Lab Tests (TST/Mantoux Test; CBC/FBC; Lymphocyte Count; ESR; Serology Test)	Microbiological Diagnostic Tests (Sputum Test; Gastric Lavage; AFB; Smear; DST; GeneXpert; PCR)	Histopathological Diagnostic Test (Biopsy)	National Scoring System (A combination of history, clinical signs and symptoms, CXR and TST)	Start Tx without Confirmation (no diagnostic tests carried out)
Indonesia (n = 53)	39	38	60	0	0	3	0
Nigeria (n = 20)	10	5	8	6	0	1	1
Pakistan (n = 35)	2	26	61	26	2	0	0
TOTAL (n = 104)	51	69	129	32	2	4	1

Contact tracing and treatment management were reported to be weak in the facilities visited in all three countries. Healthcare providers sometimes asked the caregiver or patient about history of contact with an adult having pulmonary TB, but the index case was not generally identified, there appeared to be little follow-up and isoniazid preventive therapy (IPT) prescription was not consistent.

A key challenge in the treatment of childhood TB is the lack of adherence to treatment guidelines due in part to the absence of quality-assured, appropriately dosed and formulated paediatric medicines. Medicines were generally purchased privately (as opposed to accessing NTP supplies), and consisted of a combination either of isoniazid, rifampicin, pyrazinamide (HRZ) or of isoniazid, rifampicin, pyrazinamide and ethambutol (HRZE), in powder form, fixed dose combination or as syrups. A small number of providers were still prescribing streptomycin, which is no longer recommended by WHO. Treatment was generally prescribed for 6–9 months, with an extension from 9 to 12 months if no improvement was observed, notably in Indonesia. Preference for drug formulations differed between countries, with a greater use of powder forms in Indonesia, while preference in Pakistan was fairly evenly split between loose tablets, fixed-dose combinations and syrups. Little information about medicines was available from the Nigeria study.

### Communication with the National TB Programme

In all three countries, reporting and communication between these facilities and the NTP was limited. Private sector facilities generally did not provide any data to the NTP. A small number of facilities in Indonesia and Pakistan that had been trained on DOTS did keep some TB-specific patient records, but did not forward numbers for paediatric patients to the NTP. In Nigeria very low levels of suspicion for childhood TB led to limited identification, diagnosis and recording of cases.

## Discussion

These exploratory studies add to the evidence of the large contribution by non-NTP sector facilities to the diagnosis and treatment of paediatric TB.

### Quantifying reporting gaps and the role of the Non-NTP sector

Importantly, the study provides some quantification of the additional numbers of children with diagnosed TB identified in the non-NTP sector, relative to existing national notification rates. The cases identified in just this limited number of facilities alone represent an estimated 15% of additional annualised yield of paediatric cases in Indonesia and 7% in Pakistan. The findings clearly suggest that further research efforts to identify the under-reporting of paediatric TB cases specifically within the non-NTP facilities is warranted.

The study highlights significant gaps in record-keeping and accessing records on paediatric TB cases. All children diagnosed with TB should be registered and treated using NTP standards–whether diagnosed and treated in NTP or non-NTP facilities. These standards include clear recording of treatment, side effects and outcomes of treatment. The study indicates that off of these were generally unavailable in non-NTP settings. In addition to the importance of these standards for clinical care of individual patients, good record keeping and sharing of case numbers is essential to improving data on the burden of paediatric TB. In addition, such data would provide a base for better quantification of product demand for new paediatric medicines, thereby contributing to incentives for market entry.

### Targeting future interventions by facility type

The findings indicate the presence of high volume ‘hubs’ which see large numbers of patients. The existence of categories of facilities that see a considerable proportion of paediatric cases in both Indonesia and Pakistan offers opportunities to increase dialogue with these treatment ‘hubs’. Identifying such sites can help focus efforts to review treatment protocols, encourage compliance with NTP recommendations, improve record-keeping at facility level, strengthen reporting to and links with the NTP, and serve as potential centers of excellence to expand understanding of a neglected disease such as paediatric TB. Such activities should be addressed as key interventions to improve understanding of the burden and treatment practices for childhood TB. Outreach to major treatment hubs could be achieved by building upon existing national public-private/public-public mix (PPM) activities.

### Improving diagnosis and case finding

Treatment providers in all three countries generally acknowledged challenges in diagnosing paediatric patients. The interviews suggest that although most providers were unaware of, or did not follow, national TB Diagnosis and Management Guidelines, many were keen to improve their diagnostic skills and asked for reference materials. Expanding awareness of such guidelines through training such as in-service training may also provide a relevant opening of discussion between the NTP and other providers, notably in the private sector.

The number of diagnosed cases in Nigeria stands out as significantly smaller than the numbers identified in the other two countries. Several sites seeing over 800 general paediatric patients per quarter, from a mixed socio-economic background, indicated that they did not come across any paediatric TB cases. This highlights a low index of suspicion for paediatric TB in Nigeria and indicates a likelihood of under-diagnosis. Understanding this dynamic may help the National TB and Leprosy Program to target interventions to improve diagnosis and treatment, particularly around case-finding and treatment-seeking interventions for children.

While healthcare providers in all three countries indicated awareness of the need for contact tracing, little appears to be done in non-NTP facilities in any of the countries to assess close contacts of TB patients or to trace the index case when none is reported (reverse contact tracing). Expanded PPM initiatives could explore new ways of focusing attention on contact tracing, or improving referencing to the NTP to follow up on index cases.

### Study limitations

Study limitations include the short timeframe for data collection (two weeks in total) limiting the time available for identifying facilities, and small sample size per facility type, with the potential for bias or skewing of results by outlier facilities. In the absence of detailed TB registers, information was commonly dependent on healthcare provider information and recall, carrying an inherent risk of recall bias while also limiting the survey teams’ ability to independently verify the data. While the interview method enabled us to gauge provider perceptions of treatment services, it also limited our ability to fully understand the level of under- or over-diagnosis.

As market-focused studies, health professionals were asked whether cases were treated without bacteriological confirmation (given the challenges of confirmation in children) in order to assess minimum additional market size. Respondents generally assumed that patients diagnosed with TB were started on treatment (with or without bacteriologic confirmation). However, as data were not available, and the study design did not allow for long term follow up, quantitative results on the numbers of children actually treated are not available. Any market estimate derived from this study would have to factor in an assumption of the percentage of children diagnosed in non-NTP facilities who then go on to receive treatment, and where they access the drugs. The data from such a rapid assessment do not allow us to confirm the treatment rates, or what proportion of children would get treatment from an NTP facility thus consuming medicines which are already part of the NTP quantification process.

As exploratory studies, the findings were not intended to be nationally representative, but rather to test assumptions in this under-studied field relating to the role of non-NTP providers in the diagnosis and treatment of childhood TB. Finally, as exploratory, qualitative studies, data were compared based on key themes, and statistical extrapolation was neither possible nor intended.

### Supporting market demand for new medicines

In view of the limited commercial value and current fragmentation of the market for paediatric TB medicines, improvements in understanding disease burden and market structure are important incentives for encouraging manufacturers to enter this small and highly specialized market. Improved market understanding and market data are critical to help ensure the availability of appropriately-formulated paediatric medicines for both commercial and public health interests. As indicated above, a deeper understanding of treatment dynamics (proportion of children on treatment, compliance rates, prescription patterns and sources of medication) would contribute considerable to improving market forecasts.

Expanding this approach to identify treatment hubs for paediatric TB in each country could contribute the data necessary to improve estimates of market size. An assessment of the non-NTP market from a supply perspective could also provide a complementary approach to help illuminate the size of the private sector market for childhood TB treatment.

## Conclusion

The challenge of estimating the burden of paediatric TB is receiving increasing national and international recognition. The importance of detecting and treating childhood TB is underscored in the Roadmap for Childhood TB, addressing not only public health concerns for children, but also the wider implications as childhood TB represents an indicator of TB transmission rates.

The proportion of children found in this study who were diagnosed with TB but not reported to the NTP confirms the potential significance of the non-NTP sector as a locus for treatment in certain high burden countries. The study highlights challenges in recording of treatment, side effects and outcomes of treatment in the non-NTP sector, leading to challenges in estimating disease burden. The results in Nigeria highlight the urgent need for increased training on TB diagnosis and treatment in children.

Further studies of this kind would contribute to a deeper understanding of treatment dynamics (proportion of children on treatment, compliance rates, prescription patterns and sources of medication, locus of diagnosis versus treatment) would contribute considerable to improving market forecasts and thus create incentives for manufacturers to enter the market.

This study emphasizes the need to capture diagnosis and treatment data from non-NTP facilities, notably through expanded public-private/public-public mix (PPM) outreach. Further research into the number of children diagnosed and treated in the non-NTP sector especially in the private sector, for example in treatment hubs, should be addressed as a high priority in scaling up availability of new paediatric treatments.

## Supporting Information

S1 Data Data on paediatric treatment in non-NTP facilities. Table A in S1 Data—Sites visited by type. Table B in S1 Data—Analysis of sites. Table C in S1 Data—Nr of patients seen. Table D in S1 Data—Breakdown of tests. Table E in S1 Data—Other analyses.
(XLSX)
